# An unusual presentation of gastric fistula following peptic perforation repair: A case report

**DOI:** 10.1016/j.ijscr.2019.01.032

**Published:** 2019-02-01

**Authors:** Mithilesh Kumar Sinha, Sudipta Mohakud, Tushar Subhadarshan Mishra, Apurba Barman

**Affiliations:** a Department of General Surgery, AIIMS Bhubaneswar, India; b Department of Radiodiagnosis, AIIMS Bhubaneswar, India; c Department of Physical Medicine and Rehabilitation, AIIMS Bhubaneswar, India

**Keywords:** Gastric fistula, Gastrocutaneous fistula, Peptic perforation

## Abstract

**Introduction:**

Peptic perforation repair is a common stomach surgery. This surgery has not been associated with delayed onset gastrocutaneous fistula formation. However such a complication has been reported following a variety of other stomach surgeries. We are reporting this case as it is a rare complication. Also its diagnosis and management is challenging.

**Case summary:**

We are presenting a case of peptic perforation repair where burst abdomen happened in the immediate post-operative period. The patient was put on conservative management. He responded well to it but his abdominal wound was not healing. After a wait of four months the wound was covered with a skin graft. The graft uptake was satisfactory but a discharging ulcer appeared on it. This condition persisted for one month. Finally a computed tomography Fistulogram (CT Fistulogram) was performed. It revealed an underlying complex gastric fistula. A repeat surgery was performed.

**Conclusion:**

A gastrocutaneous fistula diagnosed at sixth month following the peptic perforation repair and causing minimal discomfort to the patient is a rare presentation. The abdominal wound following the surgery was possibly not healing because of the underlying fistula.

## Highlights

• Late onset Gastrocutaneous fistulas are rare following the peptic perforation repair.
• A non-healing burst abdominal wound should be thoroughly investigated. It can be because of an underlying fistula.
• CT fistulogram is the investigation of choice to confirm the diagnosis.
• Operative intervention is difficult but the only method of cure in most of the circumstances.

## 1 Introduction

Gastric fistulas can present in various ways. Early in post-operative period they manifest as burst abdomen with copious discharge through the wound. The late manifestations can be subtle and usually present as discharge through the scar. Internal fistulas to other organs are also known to occur. Their course is unpredictable. Some heals spontaneously while others require meticulous surgery. This wide spectrum of presentation and progress should always be kept in mind. Even a small suspicion should be thoroughly investigated. It is important to reach a proper diagnosis before intervention. We are reporting a rare complication of peptic perforation repair from a tertiary care academic institute. The work has been reported in line with the SCARE criteria [1].

## 2 Case report

### 2.1 Presentation

A 30 year old male presented at the Surgery OPD with chief complaint of discharging wound in the upper part of the abdomen for 1 month. The problem started 6 months back when he underwent an emergency exploratory laparotomy at another medical college for 3 days old abdominal pain. A small peptic perforation was detected and was repaired with an omental patch. On fourth post-operative day the patient developed burst abdomen. It was managed conservatively. Over a period of time the bowel got contained and the patient was put on oral nutrition. The patient was discharged was doing fine at home. However his abdominal wound was not healing. In the fourth month it was covered with the split thickness skin graft. The procedure and the post-operative period was uneventful till one month. However in the fifth month a serous discharge from the upper part of the grafted surface was noticed. It was coming from a small ulcer and was small in amount. Over a the period of time till he presented at our OPD it remained small in output. It was managed by applying gauge pieces over the wound which has to be changed once or sometimes two to three times a day.

**Fig. 1:**
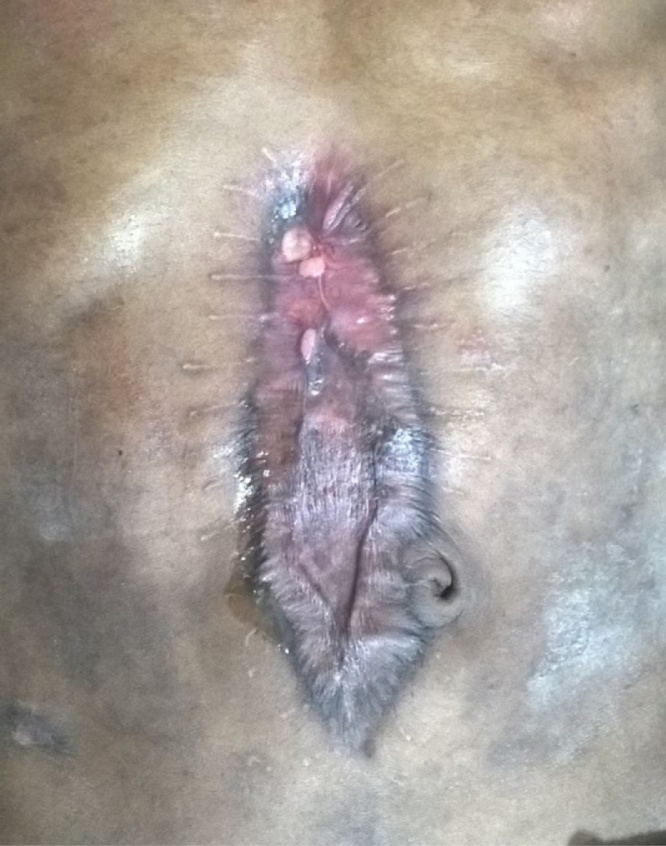
Pre-operative photograph- the uppermost depressed ulcer covered with pale granulation is the external fistula opening.

The examination of the abdomen revealed a 12 cm × 5 cm elliptical patch of skin graft over the middle of the abdomen. There was a small depressed ulcer of around 1 cm × 1 cm in its upper part covered with pale granulation showing serous ooze. Apart from this ulcer there were few other spots showing exuberant pale granulation (Fig. 1). A scar was seen at previous drain site. Palpation of the abdomen showed deficient abdominal wall below the skin graft.

### 2.2 Investigations

The blood reports were all but normal. Haemoglobin was 11.4 gm/dl with total WBC count as 11.6 × 10^3^/mm^3^. The total serum protein was 7.9 gm/dl with serum albumin as 3.5 gm/dl. A left subphrenic collection of size 8 cm × 7 cm was seen on the ultrasound. With a suspicion of some missed pathology at previous surgery site an upper GI endoscopy was performed. A small benign looking ulcer was seen at the pylorus of the stomach.

**Fig. 2:**
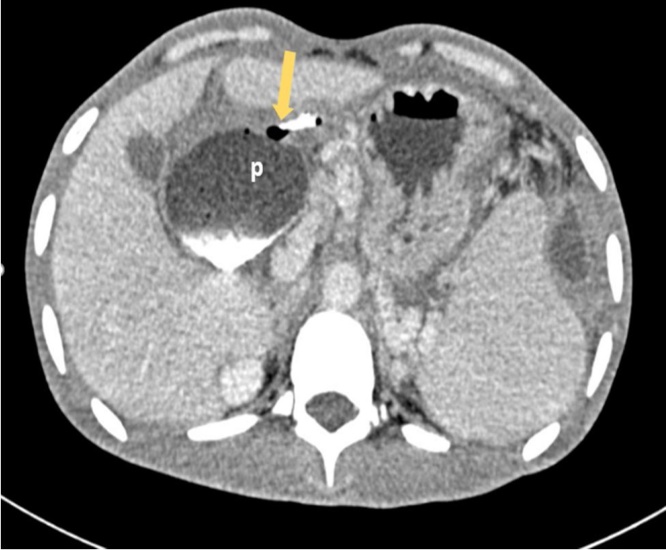
Axial CECT fistulography image shows the air and positive ionic contrast filled track (arrow) at the anterior aspect of pylorus (p) indicating the site of perforation and the enteric site of the enterocutaneous fistula.

With a strong suspicion of gastrocutaneous fistula, a CT fistulogram was performed. An enterocutaneous fistula between the pylorus of the stomach and the anterior abdominal wall was seen. A long side branch of the fistula tract was seen communicating to a moderate sized left subphrenic collection (Fig. 2).

### 2.3 Treatment

With confirmed diagnosis of complex gastrocutaneous fistula a repeat surgery was planned. The abdomen was entered after incising the previous scar on left lateral side and extending the incision downwards and upwards. Dense interbowel adhesions were encountered. Meticulous adhesiolysis and dissection was performed. The abscess cavity was drained and the perforation on the pylorus of the stomach was identified. It was around 1 cm × 1 cm size. Repeat omental patch repair was performed. In view of difficult dissection, feeding jejunostomy and retrograde tube duodenostomy were also made.

### 2.4 Post -operative period

An uneventful recovery happened. He attended follow up clinic till 4 months following the discharge. There was an incisional hernia but otherwise he was doing fine. After this he was lost to follow up.

**Timeline**

**Table:** 

Day	Event
0	Exploratory laparotomy with omental patch repair of peptic perforation
4	Burst abdomen
25	Discharged
120 (4 months)	Covered the non- burst wound with skin graft
150 (5 months)	Noticed a discharge through the graft site
180 (6 months)	Presented to OPD
182	USG abdomen done: suggestive of collection
164	Upper GI endoscopy done: Ulcer in pylorus
186	CECT abdomen done: complex fistula identified
196	Exploratory laparotomy done. Repair of the defect in stomach done
210 (7 months)	Discharged from hospital

## 3 Discussion

Clinical presentation of leaked a peptic perforation repair is usually violent. They are high output fistulas and requires good nutritional support along with control of effluents [2]. The presentation as burst abdomen alone is uncommon. In our case the burst abdomen was associated with normal wound discharge and he was doing fine on oral nutrition. These findings suggests that even if some leak happened in the post -operative period it got sealed spontaneously. This self-limiting leak explains the burst in a young person. If this hypothesis is assumed correct then a delayed fistula developed in our case and the decision to cover the burst abdomen was correct.

The only thing that does not fit well into this is the fact that the burst didn’t healed spontaneously. At fourth month it required skin grafting. A fairly large time was given for the wound to heal on its own. The built, nutrition, general condition and laboratory parameters were all favouring the healing. He was not immune suppressed or addicted to any substance. Neither he had any other co-morbidity. Non healing in such a person is difficult to explain.

The late formation of the gastric fistula is a reported event in the literature. Although never described in relation to peptic perforation, it has been described following a variety of gastric surgeries [[3], [4], [5]]. These include surgeries for diseases like gastric ulcer, gastroesophageal reflux diseases and morbid obesity. Here delayed suture line leak results in fistulas. They have also been reported following removal of gastrostomy tubes [6].

The diagnosis of gastric fistula is a challenging task. A strong clinical suspicion is necessary. Ultrasound abdomen and Upper gastrointestinal endoscopy are not diagnostic. CT Fistulogram is the investigation of choice in these circumstances [7,8].

Management of gastric fistulas is complex. Spontaneous healing sometimes happen when the fistula is simple. Presence of distal obstruction, local sepsis, multiple tracts or large defect excludes this possibility. Spontaneous healing after two months of surgery is also rare. In these circumstances surgery is the only option. The ideal time for intervention is between three to six months after the primary surgery when the patient is stable. The surgery is usually difficult because of dense adhesions. Minimal access procedures have been tried especially in bariatric and paediatric patients [3,5,9]. Certain rare fistulas to thoracic structures require specialist surgical intervention.

On retrograde evaluation the skin grafting was a controversial decision. Even though the graft uptake was near complete and patient was symptom-free for almost four weeks. A complete radiological workup including contrast enhanced CT scan could have been a more appropriate clinical decision.

## Conflicts of interest

Nil.

## Sources of funding

Nil.

## Ethical approval

Exempted from ethnical approval of our institution.

## Consent

Written informed consent was obtained from the patient for publication of this case report and accompanying images. A copy of the written consent is available for review by the Editor-in-Chief of this journal on request.

## Registration of research studies

Not applicable.

## Guarantor

Dr Mithilesh Kumar Sinha.

## Provenance and peer review

Not commissioned, externally peer-reviewed.

## CRediT authorship contribution statement

**Mithilesh Kumar Sinha:** Conceptualization, Data curation, Formal analysis, Investigation, Methodology, Project administration, Validation, Visualization, Writing - original draft, Writing - review & editing. **Sudipta Mohakud:** Conceptualization, Data curation, Formal analysis, Investigation, Methodology, Validation, Visualization, Writing - review & editing. **Tushar Subhadarshan Mishra:** Conceptualization, Formal analysis, Investigation, Methodology, Validation, Visualization, Writing - review & editing. **Apurba Barman:** Conceptualization, Formal analysis, Validation, Visualization, Writing - original draft, Writing - review & editing.

## References

[1] Agha R.A., Fowler A.J., Saetta A., Barai I., Rajmohan S., Orgill D.P., for the SCARE Group (2016). The SCARE statement: consensus-based surgical case report guidelines. Int. J. Surg..

[2] Pearlstein L., Jones C.E., Polk H.C. (1978). Gastrocutaneous fistula: etiology and treatment. Ann. Surg..

[3] Draus J.M., Huss S.A., Harty N.J., Cheadle W.G., Larson G.M. (2006). Enterocutaneous fistula: are treatments improving?. Surgery.

[4] Hollington P., Mawdsley J., Lim W., Gabe S.M., Forbes A., Windsor A.J. (2004). An 11-year experience of enterocutaneous fistula. BJS.

[5] Papavramidis S.T., Eleftheriadis E.E., Papavramidis T.S., Kotzampassi K.E., Gamvros O.G. (2004). Endoscopic management of gastrocutaneous fistula after bariatric surgery by using a fibrin sealant. Gastrointest. Endosc..

[6] Gordon J.M., Langer J.C. (1999). Gastrocutaneous fistula in children after removal of gastrostomy tube: incidence and predictive factors. J. Pediatr. Surg..

[7] Hampson F.A., Freeman S.J., Ertner J., Drage M., Butler A., Watson C.J. (2010). Pancreatic transplantation: surgical technique, normal radiological appearances and complications. Insights Imaging.

[8] Kwon S.H., Oh J.H., Kim H.J., Park S.J., Park H.C. (2008). Interventional management of gastrointestinal fistulas. Korean J. Radiol..

[9] Stringel G., McBride W., Sweny A. (2013). Extraperitoneal closure of persistent gastrocutaneous fistula in children. JSLS.

